# Intra-*FCY1:* a novel system to identify mutations that cause protein misfolding

**DOI:** 10.3389/fgene.2023.1198203

**Published:** 2023-09-06

**Authors:** N. Quan, Y. Eguchi, K. Geiler-Samerotte

**Affiliations:** ^1^ Biodesign Center for Mechanisms of Evolution, Arizona State University, Tempe, AZ, United States; ^2^ School of Life Sciences, Arizona State University, Tempe, AZ, United States

**Keywords:** protein folding, fitness competition, misfolding, yeast, deep mutational scanning, protein complementation assay (PCA), destabilizing mutations

## Abstract

Protein misfolding is a common intracellular occurrence. Most mutations to coding sequences increase the propensity of the encoded protein to misfold. These misfolded molecules can have devastating effects on cells. Despite the importance of protein misfolding in human disease and protein evolution, there are fundamental questions that remain unanswered, such as, which mutations cause the most misfolding? These questions are difficult to answer partially because we lack high-throughput methods to compare the destabilizing effects of different mutations. Commonly used systems to assess the stability of mutant proteins *in vivo* often rely upon essential proteins as sensors, but misfolded proteins can disrupt the function of the essential protein enough to kill the cell. This makes it difficult to identify and compare mutations that cause protein misfolding using these systems. Here, we present a novel *in vivo* system named Intra-*FCY1* that we use to identify mutations that cause misfolding of a model protein [yellow fluorescent protein (YFP)] in *Saccharomyces cerevisiae*. The Intra-*FCY1* system utilizes two complementary fragments of the yeast cytosine deaminase Fcy1, a toxic protein, into which YFP is inserted. When YFP folds, the Fcy1 fragments associate together to reconstitute their function, conferring toxicity in media containing 5-fluorocytosine and hindering growth. But mutations that make YFP misfold abrogate Fcy1 toxicity, thus strains possessing misfolded YFP variants rise to high frequency in growth competition experiments. This makes such strains easier to study. The Intra-*FCY1* system cancels localization of the protein of interest, thus can be applied to study the relative stability of mutant versions of diverse cellular proteins. Here, we confirm this method can identify novel mutations that cause misfolding, highlighting the potential for Intra-*FCY1* to illuminate the relationship between protein sequence and stability.

## Introduction

The majority of mutations occurring in protein-coding sequences have a high chance of destabilizing the encoded protein thus causing it to sometimes fold improperly (Pakula and Sauer, 1989). Misfolded proteins are toxic and can lead to neurodegenerative disease (Ross and Poirier, 2004), present a problem for tumor cells (Tilk et al., 2022), and can even behave as pathogens as is the case in prion diseases (Aguzzi and Calella, 2009). Natural selection to purge mutations that cause misfolding is so common that it has left a pervasive signature in genomes across the tree of life (Drummond et al., 2005; Drummond and Wilke, 2008). Quantifying the effect of mutations on protein folding and stability would offer insight into the genetic basis of misfolding-related diseases and help us predict which mutations will survive natural selection. However, current methods to quantify how mutations affect protein stability have limitations. For example, algorithms that predict how mutations affect protein stability often perform best for proteins with known structures (Pancotti et al., 2021) and fail to identify 30%–40% of mutations known to affect stability (Buß et al., 2018; Hernández et al., 2023). Alternatively, wet-lab methods are more accurate. For example, Western blot analysis of detergent-soluble (folded) and detergent-insoluble (misfolded) protein fractions has allowed for the quantification of the portion of a protein found in a misfolded state (Geiler-Samerotte et al., 2011), as well as the identification of age-related cellular processes that result in the accumulation of proteins that are prone to aggregation (Rai et al., 2021). Pulse-chase analyses to study proteins in the cellular milieu have also been used, and its integration with HaloTag labeling has enabled powerful analysis of intracellular protein stability as regulated by protein-degradation signals (Yamaguchi et al., 2009). However, these strategies are not amenable to massively parallel quantification of the effects of many mutations on protein misfolding.

High-throughput protein complementation assays (PCA) *in vivo* have been useful in higher-throughput monitoring of protein stability in the wet lab. The general PCA strategy requires that the protein of interest be fused to complementary fragments of a reporter protein. If the protein of interest folds correctly, the reporter fragments will be brought together and fold into the native structure. This reconstitutes the activity of the reporter and has measurable effects upon the cell’s phenotype, such as conferring drug resistance or exhibiting colorimetric or fluorescent signals. The two fragments of the reporter protein are dissected rationally using protein-engineering strategies, and are designed so that they cannot fold spontaneously (Johnsson and Varshavsky, 1994; Michnick et al., 2000). Cabantous et al. (2005) demonstrated this strategy in their split-GFP assay, in which the target protein is tagged with a non-fluorescent 15-amino acid fragment of GFP (GFP11) to its C-terminus. In order to complete the fluorophore formation, the other fragment of GFP (GFP1-10) must complement the GFP11 fragment fused to the target protein. In this way, the extent of protein misfolding can be correlated to cellular fluorescence intensity as measured with a flow cytometer or fluorescence plate reader. Numerous enzyme-based *in vivo* PCA assays have also been developed. The first successful enzyme-based reporter assay of protein folding was chloramphenicol acetyltransferase (CAT) (Maxwell et al., 1999), and others such as the dihydrofolate reductase (DHFR) enzyme stability assay (Tucker and Fields, 2001), complementation of β-galactosidase (Wigley et al., 2001), and the split-ubiquitin method (Raquet et al., 2001) soon followed. Similar to the split-GFP assay, the enzyme reporter activity is closely correlated to the folding of the protein of interest to which it is fused. Any misfolding of the protein of interest will prevent the two-halves of the reporter protein from coming together to catalyze its particular reaction. In general, these PCA strategies are most useful when the goal is to characterize natively folded protein variants that maintain reporter protein function [though see (Pittman et al., 2012)]. For example, Dyson et al. (2008) used the DHFR system to identify extra- and intracellular soluble expression constructs of the murine platelet endothelial adhesion molecule Pecam1 and used the constructs to generate antibodies. However, using the aforementioned PCA strategies for high throughput comparisons of mutations that result in severe misfolding is problematic because these mutations can have indistinguishably devastating effects on the function of the reporter protein. For example, in the split-GFP assay, target proteins that are misfolded will hinder self-complementation of GFP and destroy its fluorescent signal. Similarly, in the DHFR system, severely misfolded proteins will reduce DHFR activity such that cells harboring them will grow slowly and may die. Destabilizing mutations that result in misfolding may be difficult to measure or may even escape detection by these systems, thus making their characterization difficult.

In this report, we present a novel PCA strategy named Intra-*FCY1*, which is based upon the yeast cytosine deaminase protein-fragment complementation assay (Ear and Michnick, 2009). Using an insertional approach, which reduces false positives that may arise due to proteolytic cleavage or initiation of protein translation at internal sites, Intra-*FCY1* is designed for use in eukaryotic cells and utilizes the same optimized Fcy1 protein and insertion sites as described in Ear and Michnick 2009. Here, we demonstrate its usefulness for high-throughput screens of mutations to identify those that destabilize protein folding. The reason that the Intra-*FCY1* system can detect mutations that decrease protein stability is that it utilizes a toxic protein, rather than an essential protein, as a reporter. Any strains in which the toxic reporter, Fcy1, is bifurcated by a severely misfolded protein show robust *increases* in growth because the two-halves of the toxic reporter are prohibited from reconstituting. We demonstrate use of the Intra-*FCY1* system using a barcoded library of several hundred *Saccharomyces cerevisiae* strains each harboring a Cas9-edited yellow fluorescent protein (YFP) possessing a unique single amino-acid changing mutation. The mutant YFP bifurcates the toxic Fcy1 reporter. Mutations that cause YFP misfolding disrupt Fcy1 function thereby rescuing the yeast cells from Fcy1 toxicity. Using a pooled competitive growth assay, we observe that, in conditions where Fcy1 is particularly toxic, only a few of these yeast strains harboring *FCY1*-fused mutant YFPs outcompete others to rise to high frequency. We confirmed that the surviving yeast harbor misfolded mutant YFPs by Western blot analysis. Our studies provide proof of principle that misfolded proteins abrogate the toxicity of the Fcy1 protein to which they are fused, and demonstrate the potential of the Intra-*FCY1* system to identify and characterize mutations that cause protein misfolding.

## Results

### Introducing Intra-*FCY1*, a high throughput assay for studying how mutations affect protein stability

**FIGURE 1:**
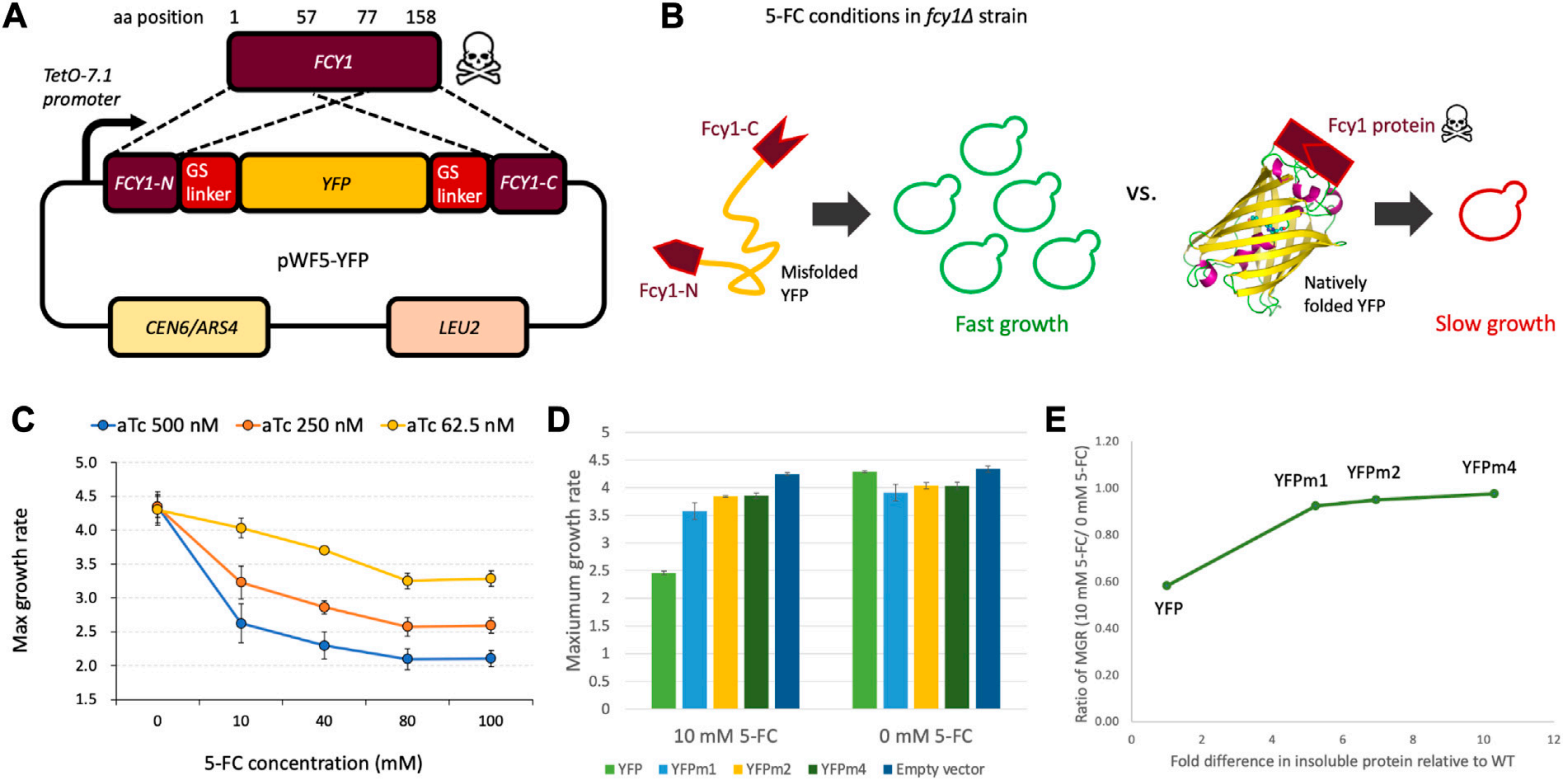
**(A)** Design of the plasmid used in the Intra-*FCY1* method, which we name pWF5. YFP was inserted between the N-terminal and C-terminal regions of *FCY1* with Glycine-Serine linkers, and the fusion gene was expressed from the tunable *TetO-7.1* promoter on a single-copy plasmid (pRS315). **(B)** Schematic of the Intra-*FCY1* mechanism. In the *fcy∆* strain in the presence of 5-FC, the growth rate depends on the folding of the inserted protein into Fcy1. **(C)** Maximum growth rate of the yeast cells harboring pWF5-YFP among different aTc and 5-FC conditions. maximum growth rate is reported in units of min^−1^ × 10^−4^. **(D)** Maximum growth rate of the yeast cells harboring pWF5-YFP, pWF5-YFPm1, pWF5-YFPm2, and pWF5-YFPm4 at aTc 500 nM in test (10 mM 5-FC) and control (0 mM 5-FC) conditions. Error bars represent standard error across 3 replicate experiments. **(E)** The horizontal axis represents the fold difference in insoluble protein relative to YFPwt as reported in Geiler-Samerotte et al. (2011). The vertical axis represents the measurements reported in panel **(D)** after normalizing maximum growth rates in the test by the control condition.

Intra*-FCY1*, a novel method designed to detect the extent of which a protein is misfolded, is based on the yeast cytosine deaminase protein-fragment complementation assay (Ear and Michnick, 2009). The metabolite produced by *FCY1*, a cytosine deaminase, is part of the pyrimidine salvage pathway and is responsible for converting cytosine to uracil. This same metabolite also has a toxic function when cells are treated with the drug 5-fluorocytosine (5-FC). In this case, Fcy1 converts 5-FC to toxic 5-fluorouridine triphosphate (5-FUTP) in a pathway that depends on Fcy1 activity (Fang et al., 2004). We hypothesized that, by inserting a target protein between the N-terminal and C-terminal regions of *FCY1* (Figure 1A), the stability of the target protein would affect the stability of Fcy1. We expressed fusion protein by promoter *TetO-7.1*, whose strength can be regulated by anhydrotetracycline (aTc) (Azizoglu et al., 2021).

The critical difference between the Intra-*FCY1* system and other chimeric systems for measuring protein stability [*e.g.*, the DHFR system (Pittman et al., 2012)], is that Fcy1 is a toxic protein in 5-fluorocytosine (5-FC) media. In the *fcy1∆* strain in 5-FC media, the growth rate of the yeast is negatively related to the activity of a Fcy1 fusion protein and its protein expression level from a plasmid. Thus, we expect that the insertion of a stably folded target protein should maintain the activity of Fcy1, thus leading to a slower growth rate. Conversely, the insertion of an unstable target protein should reduce the activity of Fcy1 and lead to a faster growth rate (Figure 1B).

By using a toxic protein as a sensor, we aim to sensitize our system to detect and more precisely quantify the relative stabilities of destabilizing mutations that cause protein misfolding. The reason is because, in our Intra-*FCY1* system, destabilizing mutations to the protein of interest are actually advantageous. This is helpful because measuring the relative fitness of strains possessing deleterious mutations in pooled fitness competitions is more error prone than measuring fitness of strains possessing advantageous mutations (Kinsler et al., 2022; Limdi and Baym, 2022). Pooled fitness competitions represent incredibly high-throughput screens of mutant strains (Venkataram et al., 2016; Kinsler et al., 2020; Kinsler et al., 2022). The Intra-*FCY1* method allows us to leverage the power of a pooled fitness competition for studying mutations that cause protein misfolding. By flipping the system such that the most misfolded protein variants result in the fastest growth (Figure 1B), we aim to enable a more precise calculation of their relative growth rates and thus relative stabilities, particularly using high-throughput pooled assays where many labeled strains are competed in the same flask (Kinsler et al., 2020; Cisneros et al., 2023).

### When bifurcated with a natively folded protein, Fcy1 decreases yeast growth rate

To test whether our system is effective, we first needed to show that bifurcating Fcy1 with a stable protein would not disrupt its toxicity. To show that stabilized Fcy1 fusion protein reduces yeast growth in the presence of 5-FC, we used yellow fluorescent protein (YFP), one of the fluorescent proteins with robust folding (Aliye et al., 2015). YFP does not have any physiological activity in yeast cells; thus, YFP function is not expected to affect yeast’s growth rate (Geiler-Samerotte et al., 2011). We inserted wildtype YFP (YFPwt) into Fcy1 to create Fcy1-fused YFP (Figure 1A) and expressed the fusion protein by the promoter *TetO-7.1*, which can be regulated by the concentration of the drug anhydrotetracycline (aTc) in the growth medium. A decreasing trend in maximum growth rate was observed as the concentration of 5-FC and aTc increased (Figure 1C). A significant decrease in maximum growth rate was observed with increasing aTc concentration in all 5-FC-added conditions. This result suggests that the expression level of the Fcy1 fusion protein in the cells is negatively correlated with the growth defect. These results indicate that stabilized Fcy1 fusion protein inhibits growth in the presence of 5-FC in a dose-dependent manner.

### The Intra-*FCY1* system recapitulates relative stabilities of model misfolded proteins

To examine whether destabilizing the Fcy1 protein by bifurcating it with an unstable protein alleviates its toxicity, we used misfolded YFP mutants named YFPm1, YFPm2, and YFPm4, which possess 4, 6, or 10 mutations, respectively. Previous work has demonstrated via Western blot that these mutant YFPs are misfolded and have decreased stability relative to YFPwt (Geiler-Samerotte et al., 2011). Therefore, we inserted YFPm1, YFPm2, and YFPm4 into Fcy1 to create 3 different Fcy1 fusion proteins (collectively referred to as Fcy1-fused YFPm’s), and expressed these under 5-FC 10 mM and 5-FC 0 mM conditions. We observed that the maximum growth rates of Fcy1-fused YFPm’s were significantly higher than that of Fcy1-fused YFPwt (Figure 1D; left) (YFPm1; *p* = 9.7E-4, YFPm2; *p* = 1.0E-4, YFPm4; *p* = 5.2E-5, Student's t-test). This result suggests that the reduction of Fcy1 stability by insertion of misfolded YFP mutants alleviates Fcy1 toxicity.

We also performed a control experiment to see if the growth defect observed in the strain harboring YFPwt is cured when we remove 5-FC from the growth medium. Under these conditions, the strain expressing the Fcy-YFPwt fusion protein did indeed recover its growth rate. In fact, the maximum growth rate of the Fcy1-fused YFPm’s were now observed to be slightly lower than that of the Fcy1-YFPwt fusion protein (Figure 1D; right). These decreases in maximum growth rate in the 5-FC 0 mM condition may be due to the inherent toxicity of the misfolded YFP mutants; they were previously shown to slightly decrease fitness when expressed without the Fcy-1 fusion (Geiler-Samerotte et al., 2011). These results suggest that two factors affect maximum growth rate of the strains expressing Fcy1-fused YFPm’s under 5-FC 10 mM conditions. First, there is the more salient increase in maximum growth rate due to the reduction of Fcy1 activity by destabilization via insertion of a misfolded protein. Second, there is a mild decrease in maximum growth rate due to toxicity of the misfolded YFP Fcy1 fusion proteins themselves. Thus, the maximum growth rates under 5-FC 10 mM (test) conditions can be normalized by the maximum growth rates under 5-FC 0 mM (control) conditions (Figure 1E; Supplementary Figure S1).

After normalization, we asked whether the Intra-*FCY1* method can recapitulate the relative stabilities of YFPm1, m2 and m4. Previous work measured the relative stabilities of these mutant YFPs using Western blot, finding YFPm1 to be the least misfolded and YFPm4 to be the most misfolded (Geiler-Samerotte et al., 2011). Our results using the Intra-*FCY1* system confirm this rank order, as the maximum growth rate increases as the severity of misfolding increases from YFPm1 to YFPm4 (Figures 1D, E). This experiment was performed using a plate reader, which has far less power to distinguish small growth rate differences between high-fitness strains than does a pooled competition assay (Kinsler et al., 2022). These results are thus suggestive that the Intra-*FCY1* method, when performed as a pooled competition assay, will be able to distinguish between misfolded mutants of differing severity. More explicitly, our results confirm that proteins with destabilizing mutations indeed have faster growth rates than their wild-type counterparts when expressed using the Intra-*FCY1* system.

### The Intra-*FCY1* system can cancel the localization of a protein of interest

The Intra-*FCY1* system uses Fcy1 as a toxic reporter, which requires that Fcy1 functions properly in the cytosol (Endo and Takahashi, 1973). Since YFP is also a cytosolic protein, it is perhaps less surprising that Fcy1 toxicity is preserved in the YFPwt-Fcy1 fusion. Previous reporter assays demonstrate that creating a fusion protein can sometimes alter the intracellular localization of a reporter, leading to false-negative/positive results (Tarassov et al., 2008; Rochette et al., 2015). For this reason, we decided to build our Intra-*Fcy1* system such that the target protein (*e.g.*, YFP) is sandwiched between two-halves of the reporter protein, Fcy1. Thus any N-terminal or C-terminal localization tags on our target protein can be canceled by hiding them within Fcy-1. Another option, not explored here, is to remove any localization signal on the protein of interest before inserting it into the Fcy1 fusion construct.

**FIGURE 2:**
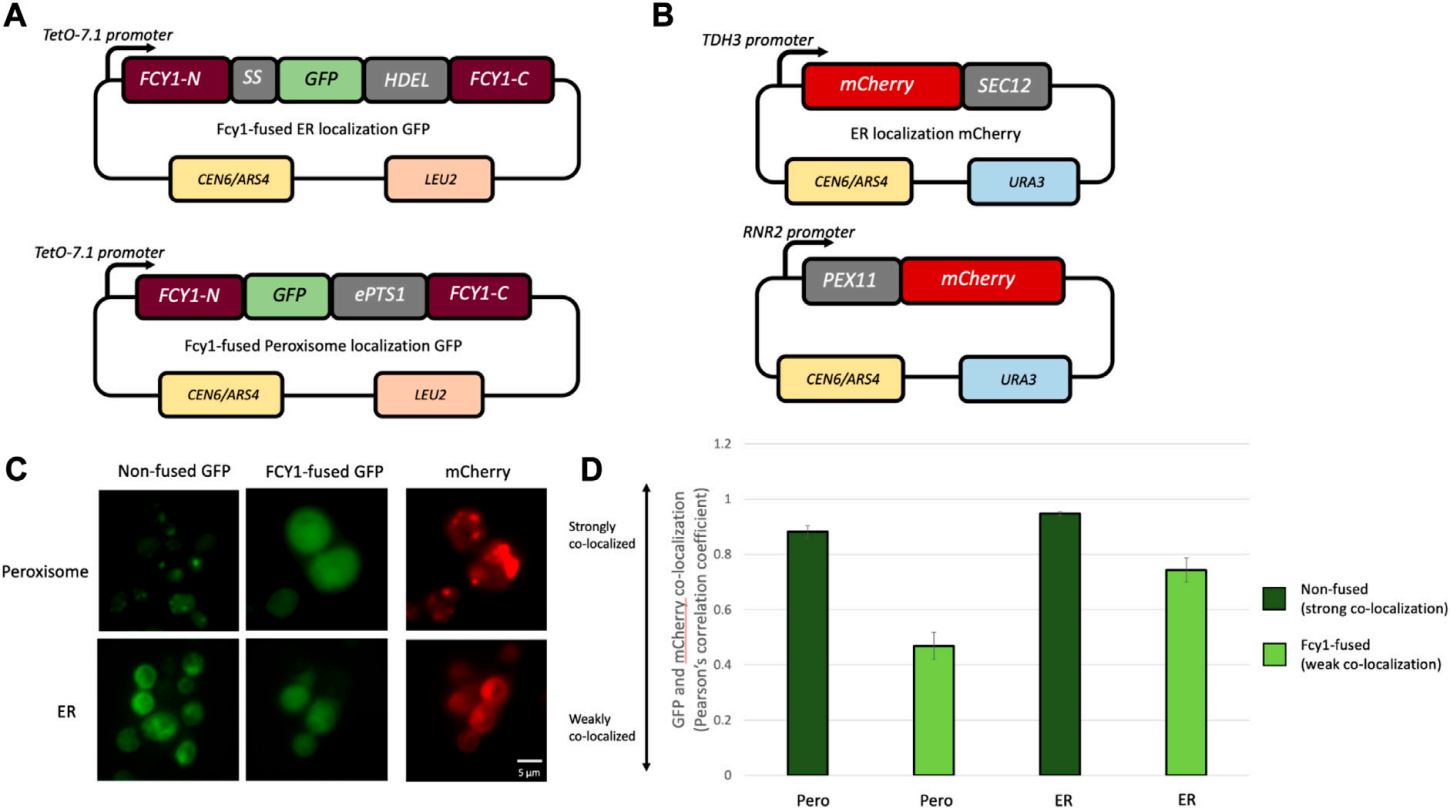
**(A)** Design of the plasmids used in measuring the localization of modified GFPs fused to Fcy1. Fcy1-fused modified GFP was expressed from the tunable *TetO-7.1* promoter on a single-copy plasmid (pRS315). **(B)** Design of the plasmids expressing mCherry fused to *SEC12* to measure ER localization or *PEX11* to measure peroxisomal localization. **(C)** Localization of Fcy1-fused and non-fused GFPs and ER- or Pero-mCherry. **(D)** Pearson correlation coefficient values between GFP and mCherry fluorescent signals for all strains. Each strain was analyzed with 2 biological replicates, with 5 technical replicates within each biological replicate. Error bars represent standard error.

To estimate the effect of intracellular localization tags of a target protein on the cytosolic localization of the Fcy1 reporter protein in our Intra-*FCY1* system, we measure the localization patterns of two Fcy1-fused modified green fluorescent proteins (GFPs) containing N-terminal localization tags; ER-localization GFP (ER-GFP) (Clayton and Mowatt, 1989; Ho et al., 2006) and peroxisome-localization GFP (Pero-GFP) (DeLoache et al., 2016) (Figure 2A). Both the ER-GFP and Pero-GFP strains also express mCherry fused to an endogenous ER- or Pero-localization marker protein. This allows us to determine where our Fcy1-fused GFPs localize by comparing green and red fluorescent signals. In the ER-GFP strain, mCherry is fused to *SEC12* (ER-mCherry), and in the Pero-GFP strain, mCherry is fused to *PEX11* (Pero-mCherry) (Figure 2B). We overexpressed these mCherry-fused proteins from the *TetO-7.1* promoter in aTc 500 nM conditions.

Our data suggest that our sandwich design cancels localization signals on the target protein. Although non-fused modified GFPs were localized to the designated subcellular compartments, Fcy1-fused modified GFPs were localized in the cytosol (Figure 2C). This confirms that our design was successful at canceling the ER and peroxisomal localization signals by sandwiching them inside the Fcy1 protein. Colocalization analysis was carried out on images taken on a Nikon Eclipse Ti2-E inverted fluorescence microscope. In order to calculate the Pearson’s correlation coefficient (PCC) describing how often mCherry and GFP colocalize for each strain, 5 regions of interest were randomly selected in 2 biological replicates and the PCC was calculated using Nikon’s NIS-Elements proprietary colocalization analysis software. While the mCherry and GFP fluorescent signals almost always colocalize in strains without the Fcy1 fusion, this correlation was weaker in strains where the GFP was sandwiched between the two fragments of Fcy1 (Figure 2D). We do not expect the PCC to approach 0 in these strains because in a two dimensional microscopy image of a three dimensional cell, regions of overlap do not always indicate co-localization. In sum, these results suggest that inserting an intracellular-localized protein into Fcy1 cancels its localization. This suggests that our Intra-*FCY1* system can be used to compare the relative stabilities of a wide variety of proteins, including those with different localization patterns.

We also measured the growth rates of strains expressing Fcy1-fused modified GFPs to confirm that, despite their localization signals, Fcy1 is localizing to the cytosol where it has toxic effects on growth. We measured the maximum growth rates of these fusion proteins when expressed at the maximum induction level from the *TetO-7.1* promoter under test (5-FC 10 mM) and control (5-FC 0 mM) conditions. The maximum growth rates of those fusion proteins in test conditions were significantly lower than those in control conditions (Supplementary Figures S2A, B), indicating that enough of the Fcy1 was present in the cytosol to cause toxicity.

### The Intra-*FCY1* system can screen large numbers of mutants for those that cause misfolding

**FIGURE 3:**
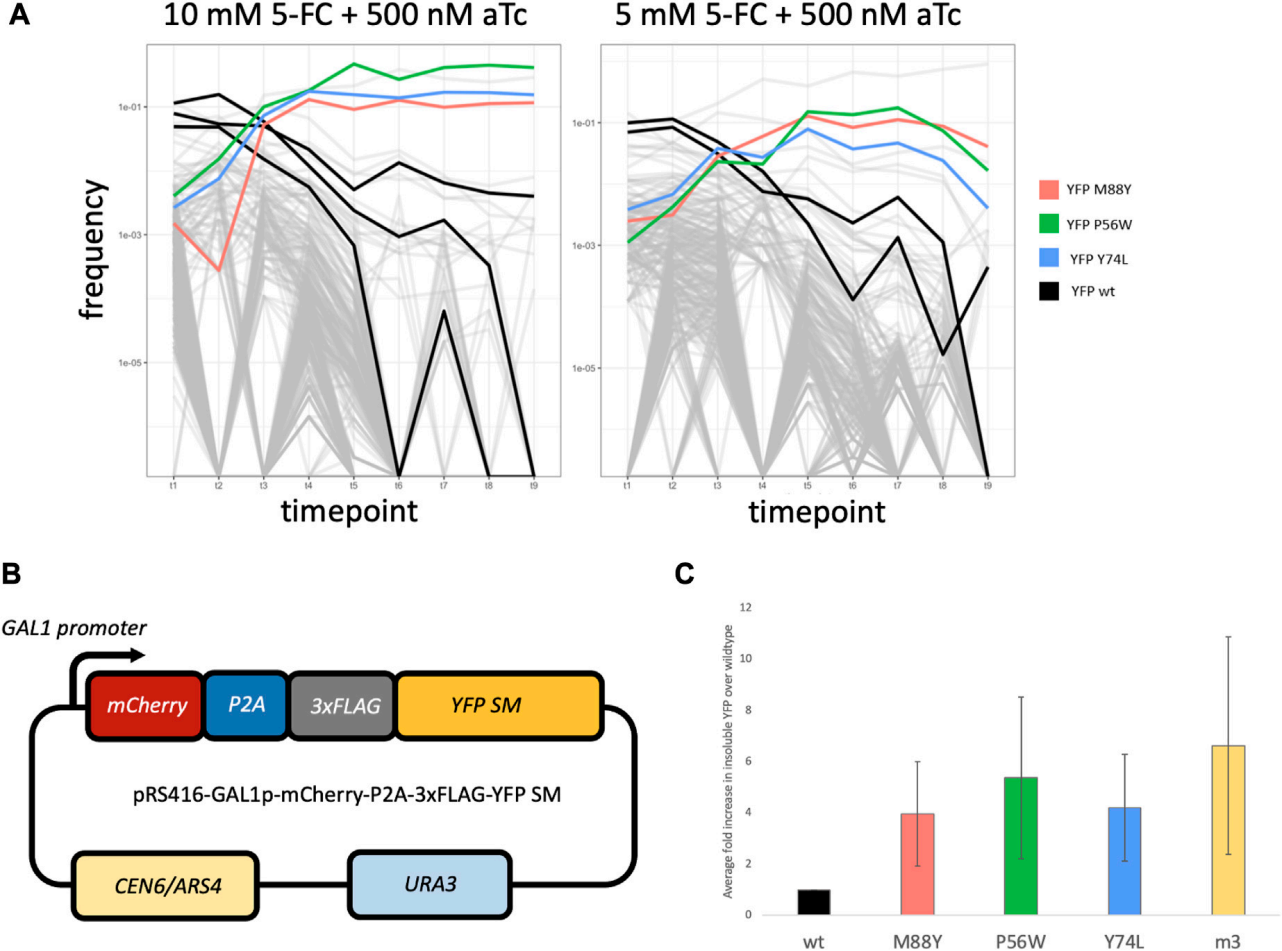
**(A)** Frequency plots for the pooled competitive growth assays in 10 mM 5-FC + 500 nM aTc (left) and 5 mM 5-FC + 500 nM aTc (right). Every line represents a yeast strain harboring a different mutant YFP within the Fcy1 protein. The vertical axis depicts the frequency of each strain’s barcode which was measured by extracting DNA from the pooled competition and sequencing the barcode region. Most strains’ barcodes decreased in frequency over time, including the baseline strains possessing wildtype YFP (highlighted in black). However, a few strains rise in frequency. Three strains that rose in frequency across both concentrations of 5-FC are highlighted in red, green and blue. These possess the mutant YFPs that are recreated in the YFP SM series. **(B)** Design of the plasmid used in constructing the YFP SM series. YFP is tagged with 3 FLAG tags and fused to mCherry, separated by a P2A self-cleaving peptide sequence. Expression of this fusion protein is controlled by a *GAL1* promoter. **(C)** Average fold differences for insoluble protein abundance from Western blotting between 3 biological replicates for YFPwt, YFP SM series, and YFPm3. Error bars represent standard error across 3 biological replicates.

We next tested whether the Intra-*FCY1* system could screen hundreds of point mutations and identify those that caused more severe misfolding. We pooled hundreds of yeast strains each expressing Fcy1 bifurcated by a YFP with a different missense mutation. Initially, we selected 105 amino acid positions that were predicted to be located in regions within the YFP structure that have a high degree of rigidity, thus ensuring a high likelihood of the protein structure being affected by mutation (Cilia et al., 2014). We used a highly efficient CRISPR system for yeast (Sharon et al., 2018) to create a mutant library, but did not recover all 105 × 19 (1,995) amino acid-changing mutants at high enough frequency to seed a pooled competition experiment. When we sequenced our starting pool at a depth of 10 M reads, we observed ∼570 mutants with 275 present at a frequency greater than 0.001 (Supplementary Material S10). In addition to strains possessing mutant YFPs, we added several strains harboring wild-type YFP to the pool to serve as a baseline. Each mutant, as well as the wild-type YFP baseline strains, possesses a DNA “barcode” that we can use to track its frequency by next-generation sequencing before, during and after the competitive growth period for a total of nine time points (Figure 3A). From these assays, our goal was to find single amino-acid changes to YFP that severely affect protein stability and folding through observing their performance in pooled competition.

We hypothesized that any strains that rise to high frequency over time, outcompeting the other strains in the pool, would harbor more severely misfolded YFP variants. This is because severely misfolded YFPs would disrupt the function of Fcy1, lessening its toxic effect on fitness. We also expected that the wild-type YFP baseline strains would be quickly outcompeted because the stably folded YFP would not abrogate Fcy1 function, thus Fcy1 would slow their growth. Indeed, this appears to be the case. We observed that three YFP mutants rose to high frequency in both high (10 mM) and medium (5 mM) concentrations of 5-FC (Figure 3A; colored lines) whereas strains harboring wild-type YFP fused to Fcy1 were outcompeted (Figure 3A; black lines). In fact, almost all of the yeast strains present were quickly outcompeted in both the 10mM and 5 mM 5-FC conditions (Figure 3A; light gray lines crash to low frequency). We did not observe this in control experiments in which the media did not contain 5-FC; in these conditions, barcode frequencies were more stable over time (Supplementary Figure S4). These results suggest that the growth advantage of the few strains that outcompete others in 5-FC containing conditions is mediated by the toxicity of Fcy1, which is abrogated in strains possessing misfolded YFPs. While the concentrations of 5-FC we chose are appropriate for distinguishing the most severely misfolded mutants from others, lower concentrations may be useful for distinguishing stabilities of more moderately misfolded mutants. In sum, our results suggest that the Intra-*FCY1* system is effective at screening large numbers of mutants in pooled cultures for those that cause severe misfolding.

### Misfolding mutations identified by Intra-*FCY1* cause YFP to localize to insoluble fraction

To test whether the three YFP mutants highlighted in color in Figure 3A are truly misfolded, we performed Western blots on the insoluble protein fraction from strains expressing each one. Then we asked whether there was more insoluble YFP present in each of these three strains than in a strain expressing wild-type YFP. This is a better approach than studying the relative fluorescence of each strain because previous work has shown that YFP fluorescence and folding are not necessarily correlated (Geiler-Samerotte et al., 2011), though we found it promising that by eye all three YFP mutants of interest fluoresced less strongly than YFPwt (data not shown). Additionally, all three mutants represent cases where either a small amino acid was replaced by a large one, or *vice versa*. These observations are suggestive that these YFP mutants are misfolded to some degree, however, there is another explanation for their increased relative fitness in our experiments: background genomic mutations. Though beneficial mutations are rare, it is possible that one occurred somewhere in the genome of each of these strains during the procedure to insert the Intra-*FCY1* plasmid. In general, there are two ways to control for background mutations. First, one could create replicate Intra-*FCY1* yeast libraries that would each suffer from different *de novo* background mutations. Then only YFP mutants that rise to high frequency in experiments pertaining to both libraries should be considered misfolding mutants. Second, one could perform Western blots on any potentially misfolded YFPs, as we do here, though this method is lower throughput.

To perform Western blots that test whether these three YFP mutants are misfolded, we first constructed three strains harboring these three YFP mutants (positions M88Y, P56W, and Y74L, hereafter referred to as YFP SM series) tagged with three FLAG tags, fused to mCherry separated by a P2A self-cleaving peptide sequence, and expressed from a galactose-inducible promoter (Figure 3B). We also constructed identical strains harboring wild-type YFP and YFPm3, which is one of four previously well-characterized misfolded YFP mutants (Geiler-Samerotte et al., 2011). We grew each of these five strains to mid-log phase while inducing YFP expression with galactose. Protein was extracted and soluble and insoluble protein fractions were prepared from all five strains, and Western blots were performed on the insoluble protein fraction to detect YFP. We performed these experiments in triplicate.

Our hypothesis was that misfolded proteins would be found in the insoluble fraction, therefore, YFPm3 and all three proteins in the YFP SM series would be detected in greater abundance in the insoluble fraction than would be wild-type YFP. Indeed, this is what we observed (Figure 3C). We used FIJI (Schindelin et al., 2012) to quantify the abundance of insoluble YFP in all strains. Fold differences for each YFP mutant was then calculated relative to wildtype, thus indicating the degree of misfolding in each YFP mutant. After averaging across all three replicates, we found that the three strains expressing the YFP SM mutants all had significantly more misfolded YFP in their insoluble fraction than did the strain expressing wild-type YFP (Figure 3C). These results confirm that the Intra-*FCY1* method can successfully identify mutations that cause protein misfolding. Full Western blot images, including total protein (a loading control) and mCherry blots (an expression control) can be found in Supplementary Figures S5, S6.

Taken together, these results are promising in terms of the usefulness of the Intra-*FCY1* system to identify misfolded protein variants. We have demonstrated that Intra-*FCY1* can distinguish misfolded from stably-folded protein variants when strains possessing those variants are grown independently in 96-well plates (Figure 1) or in a pooled fitness competition monitored via next-generation sequencing of DNA barcodes (Figure 3). We have also confirmed the accuracy of Intra-*FCY1* in two separate ways, first by using known misfolded variants (Figures 1D, E) and then by follow-up experiments on novel misfolded variants to show they are indeed misfolded (Figure 3C). Finally, we have demonstrated the potential of this system for use with a broad variety of proteins by showing its effectiveness in proteins with different localization patterns (Figure 2). These experiments demonstrate the potential utility of the Intra-*FCY1* system for revealing new insights about changes at the DNA sequence level that cause protein misfolding.

## Discussion

Protein misfolding is a root cause of many biological problems and a better understanding of the relationship between protein sequence and stability is needed. For example, protein misfolding is a key histopathological characteristic of many diseases like ALS, Parkinson’s, and Alzheimer’s. However, the mutations that cause misfolding are unknown in many cases, which limits our understanding of the mechanistic basis of disease and makes disease incidence difficult to predict (Hardy, 2001; Hofmann et al., 2019; Islam et al., 2021). Unfortunately, many high-throughput assays that survey the effects of mutations on protein stability are higher powered to study mutations that increase stability, rather than destabilizing mutations such as those that may be associated with disease. The Intra-*FCY1* system, by allowing strains harboring misfolded proteins to outcompete those harboring stably folded variants, overcomes this limitation and offers new possibilities. For example, perhaps this system, if applied broadly, could improve predictions of protein stability from sequence by defining general properties of destabilizing mutations, such as where they tend to be located within a protein structure and which amino acid changes they tend to involve.

A system for identifying or predicting protein misfolding may be useful for many reasons outside of determining the genetic changes that cause human disease (Eguchi et al., 2019). Such a system could be used to learn basic cell biology. For example, protein misfolding is a problem across the tree of life. Many different refolding or degradation pathways exist to manage this burden. One way to gain insights about these refolding mechanisms and degradation pathways is to generate synthetic proteins with different degrees of misfolding (Geiler-Samerotte et al., 2011; Geiler-Samerotte et al., 2013; Shin et al., 2021). Generating collections of misfolded proteins has also been useful in quantifying the cost of misfolding on cell fitness and understanding the mechanistic basis of this cost (Geiler-Samerotte et al., 2011; Bershtein et al., 2012; Kintaka et al., 2016). All of these research goals rely on first identifying mutations that will lead to misfolding of a protein of interest.

Protein misfolding is also an important factor in cancer evolution because the high mutation rate of tumors causes them to have a high burden of misfolded proteins (McFarland et al., 2013). Quantitative information about the misfolded protein burden of a tumor and how that burden correlates with its proliferation rate could lead to new treatment possibilities. Understanding more about the refolding and degradation pathways a cell uses to contend with misfolded proteins could similarly yield new therapeutic targets (Tilk et al., 2022). Given that next-generation sequencing of tumors is rapidly becoming more common, a method to infer a cell’s misfolded protein burden from its genome may also be useful. All of these goals are served by a system that identifies and helps us learn more about mutations that destabilize protein folding.

Many massively parallel growth competition assays for measuring the impacts of mutation on cell fitness have been developed (Fowler and Fields, 2014; Venkataram et al., 2016; Sharon et al., 2018; Kinney and McCandlish, 2019; Flynn et al., 2020; Kinsler et al., 2022). These types of incredibly powerful assays can be constructed such that they compare how mutations affect protein stability; however, many such screens are higher powered to study mutations that improve stability (Dyson et al., 2008; Foit et al., 2009; Traxlmayr et al., 2012; Kim et al., 2013; Motiwala et al., 2021). These assays are not optimal for studying mutations that cause misfolding, as misfolding-causing mutations tend to decrease in frequency throughout the course of competition experiments, making them less tractable. Massively parallel fitness competition experiments are more error prone when it comes to studying mutations that cause decreases in a strain’s frequency relative to other strains (Limdi and Baym, 2022; F; Li et al., 2018; Kinsler et al., 2022).

Here we offer a potential solution: the Intra-*FCY1* system. This system turns the tables of the growth competition such that strains expressing misfolded proteins rise to high frequency. We have demonstrated proof in principle that such strains do indeed rise to high frequency in competition experiments utilizing Intra-*FCY1* to survey hundreds of mutations to a model protein. We have also shown that Intra-*FCY1* may be broadly applicable to target proteins with non-cytosolic localization because it cancels organelle localization. However, there are limitations to Intra-*FCY1*, including mutations and proteins that we may not be able to study with this system. For example, mutations that cause rapid degradation may rise to high frequency by causing concomitant degradation of the Fcy1 protein, even if they do so without destabilizing the protein of interest. Also, in special cases, one of the Fcy1 fragments may be sterically occluded in the folded fusion protein, thereby hindering proper Fcy1 function even for correctly folded proteins. And the folding of the protein of interest may be adversely affected in cases where Fcy1 fusion directs proteins that usually localize elsewhere to the cytosol. Finally, special care must be taken when studying endogenous proteins to choose experimental conditions in which compromising the function of the protein of interest does not affect fitness, or confirming that expression of the Fcy1-fused copy does not interfere with the function of the natively expressed copy. All of these limitations are not unique to Intra-*FCY1* but are also limitations of other protein complementation assays.

A potential strength of the Intra-*FCY1* system for future study is that it is tunable. For example, we show that using a lower concentration of inducer reduces the toxicity of FCY1-fused YFPwt (Figure 1C). Thus, by using different concentrations of inducer (anhydrotetracycline) and drug (5-fluorocytosine), it may be possible to fine-tune the Intra-*FCY1* system to focus on precisely quantifying the effects of milder misfolding-causing mutations, in addition to using the system to identify more severely destabilizing mutations like those reported here. We caution against using experiments performed in a plate reader to select drug and inducer concentrations for use in a massively parallel pooled fitness competition, as we have observed that strains with discernible fitness disadvantages as measured by a plate reader (YFPwt in Figure 1) are very quickly outcompeted in a fitness competition (YFPwt in Figure 3). In addition to drug and inducer concentration, the precision with which we can distinguish the effects of mutations on protein stability using massively parallel growth competitions depends on how deeply we sequence the barcode associated with each mutation. Given that sequencing costs are decreasing, and that designing and analyzing the data resulting from massively parallel growth competitions is an active area of research (Kinsler et al., 2022; Johnson et al., 2023; Li et al., 2023), it is likely that the precision and throughput of these types of experiments will continue to improve. This may help expand the utility of the Intra-*FCY1* system to identify mutations with milder effects on stability. Given this potential, plus previous work showing that protein complementation assays are compatible with many full-length protein sequences (Mansell et al., 2008; Ear and Michnick, 2009; Foit et al., 2009; Mansell et al., 2010), the Intra-*FCY1* system is primed to allow for new insights into the relationship between a protein’s sequence and its stability.

## Materials and methods

### Strains, growth conditions, and yeast transformation

All strains used in all experiments are listed in (Supplementary Material S1).

Yeast culture and transformation were performed by the previously described methods (Amberg, Burke, and Strathern, 2005). A synthetic complete (SC) medium without uracil (-U) and/or leucine (-L) was used for yeast culture to maintain plasmids that utilize URA3 and Leu2 markers. Anhydrotetracycline (Cayman Chemicals, 10009542) was prepared as a 0.2 mM stock solution in DMSO, diluted in DMSO, and added to medium with the indicated aTc concentrations. 5-FC (Cayman Chemicals, 11,635) was added directly to the medium with the indicated 5-FC concentrations.

### Plasmids

All plasmids used in all experiments are listed in (Supplementary Material S2).

### Measurement of growth rate with a microplate reader

To generate data described in Figures 1C–E; Supplementary Figures S1, S2, cells were pre-cultured for 48 h at 30°C in media in a 96-well plate and then transferred to a new medium in a new 96-well plate. Cellular growth was measured every 30 min in OD595 for 50 h using Epoch 2 Microplate Spectrophotometer (Agilent). Maximum growth rate was calculated by the previously described method (Moriya, Shimizu-Yoshida, and Kitano, 2006). This was conducted for 2 replicates per strain. Reported maximum growth rates represent the average across all replicate wells.

### Microscopy and colocalization analysis

To generate data described in Figures 2C, D, cells were pre-cultured overnight in SC-LU media with 500 nM aTc. The cells were then diluted back and grown to log phase in the same media. Cell images were acquired using the Nikon Eclipse Ti2-E Inverted Fluorescence Microscope (Nikon Instruments) at ×60 magnification. GFP and mCherry fluorescence was detected with the FITC and TRITC channels, respectively. Colocalization analyses were performed using Nikon’s NIS-Elements acquisition and analysis software. All calculations are listed in Supplementary Material S45A, B.

### Intra-*FCY1*-YFP library design and preparation

We designed and ordered a library of guide/donor pairs for CRISPR from Twist Bioscience (San Francisco, CA) following previous work (Sharon et al., 2018). This library included guide/donor pairs targeting 105 amino acid positions in YFP that were predicted by Dynamine, a protein structure prediction software, to have a high degree of rigidity (full guide-donor sequence list can be found in Supplementary Material S7). Since we later use these guide/donor pairs as barcodes to identify strains, and since many of these guide/donor pairs only differ by a single nucleotide, we shifted their length and position so that we could more easily distinguish them from one another. We ligated these guide/donor sequences into a modified version of the pZS165 yeast shuttle vector used in previous work (Sharon et al., 2018), generously shared by Shi-An Anderson and Hunter Fraser (see Supplementary Material S2). Then we transformed this plasmid library into a yeast strain possessing a Cas9, also generously shared by the Fraser lab. Next, we transformed this library with the plasmid used in the Intra-*FCY1* method, which we named pWF5. But in future implementations, we recommend doing the preceding two steps in reverse order, first transforming a single strain with the *FCY1*-fusion plasmid (pWF5), and then transforming this strain with the guide/donor library. We believe that this will improve the diversity of guide/donors in the final yeast library. After transforming strains with both plasmids, we initiated gene-editing via the CRISPEY system by growing strains in galactose as was done previously (Sharon et al., 2018). Since there is no YFP in the yeast genome, only the copy on the *FCY1*-fusion plasmid is edited. This gene-editing system has been reported to be highly efficient, successfully editing over 95% of all strains (Sharon et al., 2018). When we PCR amplified the YFP from a small set of our engineered strains, we confirmed that all 8 with complete guide/donor sequences had successfully edited the YFP in the FCY1-fusion plasmid. In our final library, we sequenced the guide/donor pairs at a depth of 10 M reads to determine how many unique strains were present. In this pilot experiment, we found only 570 unique guide/donor pairs were present and only 275 of those were present at a frequency of 0.001 or greater (Supplementary Material S10). We believe we lost diversity when we transformed a yeast library containing thousands of different guide/donor pairs with the *FCY1*-fusion plasmid. As mentioned earlier, this would likely be solved by changing the order in which the plasmids are transformed into yeast. For this study, where our goal was to test whether the Intra-*FCY1* system could screen for mutations that cause misfolding, 275 guide/donor pairs was sufficient.

### Intra-*FCY1* competitive pooled growth assay

For the competitive growth assay, SC-HLU media was prepared containing the various concentrations of 5-FC and aTc as described in (Supplementary Material S3). Samples for each condition were prepared as described in (Supplementary Material S3), which yielded a pool composed of 50% strains harboring wildtype YFP fused to Fcy1 as a baseline and the remainder harboring the Fcy1-YFP mutants. Pooled competitive growth was carried out in L-shaped glass tubes in an Advantec Bio-Photorecorder rocking incubator (Model TVS062CA). Each sample was cell-counted using a Beckman-Coulter Z-Series Cell Counter and diluted back every 24 h for a total of 8 rounds of growth, thus yielding 9 sample timepoints. Cells from each timepoint were frozen in 10% DMSO in both a cryotube for long term storage and a 1.5 mL tube for barcode extraction and sequencing.

### Plasmid extraction, PCR amplification of the barcode, and NGS sequencing

In order to count the relative frequencies of each strain and how they changed over time, the unique guide/donor region from each strain (its barcode) needed to be prepared for sequencing. To do so, each frozen yeast sample was thawed at room temperature and was centrifuged at 15,000 rpm for 1 min. After removal of the supernatant, 250 µL of yeast lysis solution 1 (0.1 M Na_2_EDTA, 1 M sorbitol, pH 7.5) and 1 µL of Zymolyase at 5U/μL (Zymo Research, E1005) were added to the pellet. The sample was incubated at 37°C for 30 min. After incubation, 250 µL of solution 2 (0.2 M NaOH, and 1% SDS) was added to the lysed sample and vortexed. Then, 250 µL of solution 3 (8.7% acetic acid and 5 M potassium acetate) was added and vortexed. After vortexing, the sample was centrifuged at 15,000 rpm for 10 min 750 µL of the supernatant was transferred to the spin column included in Monarch Plasmid Miniprep Kit (New England BioLabs, T1010L), and the column was centrifuged at 13,000 rpm for 1 min. After discarding the flow-through, 200 µL of Plasmid Wash Buffer 1 included in the kit was added to the column, and the column was centrifuged at 13,000 rpm for 1 min. After discarding the flow-through, 400 μL of Plasmid Wash Buffer 2 included in the kit was added to the column, and the column was centrifuged at 13,000 rpm for 1 min. After discarding the flow-through, the column was spun at 13,000 rpm for 1 min for the removal of wash buffer completely. The column was inserted into a new 1.5 mL tube, and 30 µL of DNA Elution Buffer included in the kit was added to the center of the matrix on the column. After waiting 1 min at room temperature, the tube was centrifuged at 13,000 rpm for 1 min to elute plasmids. The concentration of the plasmid was quantified by using Qubit dsDNA HS Assay Kit (Thermo Fisher Scientific, Q32854) on Qubit 4 Fluorometer (Thermo Fisher Scientific, Q33226).

PCR amplification of the barcode was performed by a two-step PCR scheme that controls for PCR duplicates as well as index swapping, very similar to the protocol described in (Levy et al., 2015; Kinsler et al., 2020). The forward and reverse primers which were used in the first PCR each had a unique 8-mer index for multiplexing in downstream analysis. The indexes were selected by using BARCOSEL (Somervuo et al., 2018), a tool for selecting an optimal barcode, from the set of 288 barcodes prepared in (Levy et al., 2015) that allows for one nucleotide mismatch among the indexes. Another random 6-mer sequence was included in these primers to be used as a unique molecular identifier (UMI) to exclude PCR duplicates in downstream analysis. All primers in the first PCR were purified by HPLC to ensure the correct length. For the second PCR, IDT for Illumina DNA/RNA UD indexes SetA (Illumina, 20026121) and SetB (Illumina, 20026930) were used. All primers used in PCR amplification of the barcode were listed in Supplementary Material S4.

For the first step of the two-step PCR, the one reaction consisted of 13 µL of Nuclease free H_2_O, 10 µL of an extracted plasmid containing about 20 ng, 1 µL each of 10 µM forward and 10 µM reverse primer, and 25 µL of Hot Start Taq 2x Master Mix (New England BioLabs, M0496L). The first PCR was performed in hot-start PCR following the cycles: 1 cycle for 10 min at 94°C, 3 cycles for 3 min at 94°C; 1 min at 55°C; 1 min at 68°C, 1 cycle for 1 min at 68°C, and hold at 4°C. After the first PCR, the PCR product was cleaned up by using Monarch PCR and DNA Cleanup Kit (New England BioLabs, T1030L) following the manufacturer’s protocol, and the cleaned-up PCR product was eluted in 22 µL.

For the second step of the two-step PCR, the one reaction consisted of 14.5 µL of Nuclease free H_2_O, 20 µL of a cleaned-up PCR product, 10 µL of 5x Q5 Reaction Buffer, 2 µL each of forward and reverse primer of Illumina index primers, 1 µL of 10 mM dNTPs (Thermo Fisher Scientific, 18427088), 0.5 µL of Q5 Hot Start High-Fidelity DNA Polymerase (New England BioLabs, M0493L). The second PCR was performed in hot-start PCR following the cycles: 1 cycle for 30 s at 98°C, 2 cycles for 10 s at 98°C; for 20 s at 69°C; for 30 s at 72°C, 2 cycles for 10 s at 98°C; for 20 s at 67°C; for 30 s at 72°C, 20 cycles for 10 s at 98°C; for 20 s at 65°C; for 30 s at 72°C, 1 cycle for 3 min at 72°C, and hold at 4°C. The whole PCR product was loaded onto 2% of NuSieve 3:1 Agarose (LONZA, 50,090), and the band between 300 bp and 400 bp were sliced. The selected PCR product was extracted by Monarch DNA Gel Extraction Kit (New England BioLabs, T1020L) following the manufacturer’s protocol, and the extracted PCR product was eluted in 10 µL. The concentration of the product was quantified by using Qubit dsDNA HS Assay Kit on Qubit 4 Fluorometer.

The resulting samples were merged such that no two had similar Illumina or internal 8-mer indices, following a scheme to exclude any index swapping events that happened during NGS sequencing (Kinsler et al., 2020; Kinsler et al., 2022). Samples were sequenced on either a Novoseq or a Hiseq X to a coverage of an average 3.3 × 10^7^ per sample. Since these amplicons libraries have low diversity, we spiked in 20% genomic DNA to all sequencing runs.

### Processing of NGS sequencing data

NGS sequencing data were demultiplexed into mate-pair files, a forward mate read1 (R1) file and a reverse mate read2 (R2) file, by Illumina sequencer software following an i5 and i7 indexes in an Illumina adaptor sequence. To exclude PCR duplicates in downstream processing, the UMIs of R1 and R2 files were extracted by using UMI-tools (Smith, Heger, and Sudbery, 2017) with the following UMI-tools commands; umi_tools extract -I “R1 file” --bc-pattern = NNNNNN -S “extracted R1 output file” --read2-in = “R2 file” --bc-pattern2 = NNNNNN --read2-out = “extracted R2 output file.”

Then, the extracted R1 and R2 files were demultiplexed. We trimmed the 5′end region containing the index by using FLEXBAR (Dodt et al., 2012; Roehr et al., 2017) with the following FLEXBAR commands; flexbar -r “extracted R1 file” -p “extracted R2 file” -b “index FASTA file for R1” -b2 “index FASTA file for R2” -bt LEFT -be 0.125 -n 10.

The STAR index files were generated from YFP reference sequences using the STAR aligner (Dobin et al., 2013) with the following STAR commands; STAR --runMode genomeGenerate --runThreadN 10 --genomeDir “STAR index output directory” --genomeFastaFiles “reference sequence FASTA file” --genomeSAindexNbases 8.

The reads in the demultiplexed R1 and R2 files were aligned to the STAR index sequences with the following STAR commands; STAR --genomeDir “STAR index output directory” --readFilesIn “demultiplexed R1 file” “demultiplexed R2 file” --runThreadN 10 --outSAMtype BAM Unsorted --peOverlapNbasesMin 62 --peOverlapMMp 0 --outFilterMultimapNmax 1 --outFilterMismatchNmax 0 --alignEndsType EndToEnd --alignIntronMax 1 --alignIntronMin 2 --scoreDelOpen −10000 --scoreInsOpen −10000 --outFilterMatchNmin 137 --alignSoftClipAtReferenceEnds No --outReadsUnmapped Fastx.

The generated aligned sequence BAM file was sorted and indexed by using SAMtools (H. Li et al., 2009) with the following SAMtools commands; samtools sort -@ 8 -o “sorted output BAM file” “unsorted output BAM file,” samtools index “sorted BAM file.”

The duplicated reads in the indexed BAM file were excluded by using UMI-tools with the following UMI-tools commands; umi_tools dedup -I “indexed BAM file” --paired -S “output BAM file without duplicated reads” --chimeric-pairs = discard --unpaired-reads = discard --method cluster. Some small number of samples that received very high sequencing coverage took a very long time (days) to run using this method, presumably due to saturation of UMIs. Therefore we ran these using the same method but with the percentage rather than cluster method selected in the UMI-tools software.

The mapped reads in the BAM file without duplicated reads were counted by using SAMtools with the following SAMtools commands; samtools index “BAM file without duplicated reads,” samtools idxstats “indexed BAM file without duplicated reads” > “indexed SAM file without duplicated reads.”

### Severely misfolded YFP strain series construction (YFP SMs)

The severely misfolded YFP strain series (referred to as YFP SM series) were generated by transforming strains with a plasmid pRS416-GAL1p-mCherry-P2A-3xFLAG-YFP, which contains a *GAL1* promoter controlling expression of a mCherry-YFP fusion protein separated by a P2A self-cleaving peptide sequence. The YFP sequence contains 3 identical FLAG tags in tandem. The 3 strains in this series each were generated with a specific amino acid substitution in YFP on the plasmid: M88Y, P56W, or Y74L. These amino acid substitutions were generated from the pRS416-GAL1p-mCherry-P2A-3xFLAG-YFP plasmid by PCR using the primers listed in Supplementary Material S4, and forward and reverse fragments for three plasmids were created:

pRS416-GAL1p-mCherry-P2A-3xFLAG-YFP_M88Y, pRS416-GAL1p-mCherry-P2A-3xFLAG-YFP_P56W, pRS416-GAL1p-mCherry-P2A-3xFLAG-YFP_Y74L.

These fragments were integrated and each plasmid was electroporated into EnduraTM ElectroCompetent *E. coli* cells using the Bio-Rad GenePulser XCell as described in the NEBuilder HiFi DNA Assembly Electrocompetent Transformation protocol. Each plasmid was extracted using the alkaline lysis method (Birnboim and Doly, 1979) and sequence-confirmed in triplicate using Sanger sequencing (primers are listed in Supplementary Material S4). Each plasmid was transformed into yeast strain *BYW2rpn4∆ (MATa his3Δ1 leu2Δ0 met15Δ0::MET15_PRNR2_TetR-NLS-TUP1 PtetO7.1_TetR-NLS ura3Δ0)* on SC-U plates. Successful transformants were PCR-confirmed. This strain has the *rpn4* deletion which compromises its proteasome’s ability to degrade misfolded proteins. This was done to enable quantification of misfolded proteins following previous work (Geiler-Samerotte et al., 2011).

### Soluble and insoluble protein isolation

Cells were grown overnight in 10 mL non-inductive media (SC-U +2% glucose), then diluted back and grown in inductive media with a proteasome inhibitor (SC-U + 2% sucrose +1% raffinose +0.5% galactose +100 µM bortezomib (Selleck Chemicals, S1013) until the culture reached saturation. The cells were then diluted back once more and grown for an additional day in fresh inductive media overnight again with a proteasome inhibitor. The cells were then diluted back once more and grown for ∼4 h in fresh inductive media and a proteasome inhibitor. We used microscopy to confirm that cells at this stage were expressing YFP. The proteasome inhibitor was used to prevent cells from degrading the misfolded YFP that we wanted to quantify. The cells were then collected and washed twice before being resuspended in cold soluble fraction buffer (50 mM Tris-HCl (Fisher Bioreagents, BP1757-500, 1 M), 150 mM NaCl (Sigma, P9541-500G), 1% TritonX-100 (Sigma-Aldrich, T8787, 50 mL), 2 mM Ultra-Pure EDTA (Invitrogen, 15575-038), sterile water, Halt protease inhibitor cocktail (Thermo, 78429, 100X) 1:100) in FastPrep 2 mL Lysing Matrix Tubes. 200 uL of acid-washed Sigma glass beads were added to each tube and the samples were lysed in a pre-cooled MP Biomedicals™ FastPrep −24™ Classic Instrument at speed 6, 60 s per cycle, 7 cycles total. The suspension for each sample was collected and centrifuged gently for 3 min at 3,000 rpm to separate out the cell debris, and the pellet was discarded and the supernatant was collected. The supernatant was centrifuged 20 min at 15,000 rpm to fractionate the protein in the samples. 30 µL of the supernatant, containing the soluble protein fraction, was collected and resuspended in 22 µL 1X NuPAGE LDS Sample Buffer (Thermo, NP0008) for SDS-PAGE. The pellet, containing the insoluble protein fraction, was rinsed twice, then resuspended in 20 µL 1X LDS buffer. Both soluble and insoluble protein fractions for each sample were then boiled at 70°C for 10 min, centrifuged for 3 in at 15,000 rpm, then preserved at −20°C.

### Protein analysis via Western blot

#### SDS-PAGE

Each sample underwent a labeling reaction using the EZLabel FluoroNeo kit (Atto, WSE-7010) at 95°C for 3 min according to the manufacturer’s instructions. The protein marker (Precision Plus Protein Dual Color Standards, Biorad, #1610374) also underwent the same labeling reaction. 20X NuPAGE MOPS SDS Running Buffer (Thermo, NP0001) was diluted to 1X concentration with deionized water and was used to fill a Mini Gel Tank (Thermo). All samples were loaded onto a NuPAGE™ 4%–12%, Bis-Tris, 1.0 mm, Mini Protein Gel, 12-well (Thermo, NP0322BOX) using Prot/Elec Tips (Biorad, #2239917EDU) for SDS-PAGE. The gel was run at 200 V for 1 h, then the gel was imaged for total protein abundance using the AZURE 600 Western blot Imager.

#### Non-electrophoretic protein transfer and Western blotting

The gel was sandwiched between two PVDF membranes (iBlot 2 Transfer Stacks, PVDF, mini [Thermo, IB24002)] and two sheets of filter paper. All the components were soaked in PBS buffer (Phosphate buffered saline tablet [Sigma, P4417-50TAB) 5 tablets, 1 L deionized water] and placed between two glass plates. Pressure was applied overnight in order to transfer the proteins in the gel onto the two membranes. Protein transfer was deemed complete when all of the dye from the marker had disappeared from the gel and appeared onto both membranes. Both membranes were then washed twice in PBST buffer (Phosphate buffered saline tablet (Sigma, P4417-50TAB) 5 tablets, 1 L deionized water, 1 mL Tween 20 (MilliporeSigma, P9416-50 ML)) and then incubated with agitation in PBST + skim milk powder (MilliporeSigma, 1153630500) for 1 h at room temperature. Both membranes were then washed twice in PBST buffer and then one membrane was incubated with agitation in 1:2,000 ANTI-FLAG M2 monoclonal antibody (Sigma, F1804-200UG) in PBST buffer for YFP detection, and the other membrane was incubated with agitation in 1:2,000 Anti-mCherry monoclonal antibody (clone 1C51, MilliporeSigma, MAB131873) in PBST buffer for mCherry detection for 1 h. Both membranes were then washed in PBST buffer three times, then incubated with agitation in 1:10,000 N-Histofine Simple Stain Rat MAX PO (MULTI) (Nichirei Bioscience, 414191F) for 1 h. Both membranes were then washed in PBST buffer three times and imaged using the ChemiBlot setting on the AZURE 600 Western blot Imager using the SuperSignal West Femto Maximum Sensitivity Substrate (Thermo, 34094). Protein bands were analyzed using FIJI (Schindelin et al., 2012). All protein band density and fold change calculations are listed in Supplementary Material S6.

## Data Availability

The original contributions presented in the study are included in the article/Supplementary Material, further inquiries can be directed to the corresponding author.
