# Supplementary material for: Intra-FCY1: a novel system to identify mutations that cause protein misfolding
Source: Front Genet. 2023 Sep 6;14:1198203. doi: 10.3389/fgene.2023.1198203 (PMC10512024; doi:10.3389/fgene.2023.1198203)
Supplement: Supplementary file 12 [file Image1.pdf]

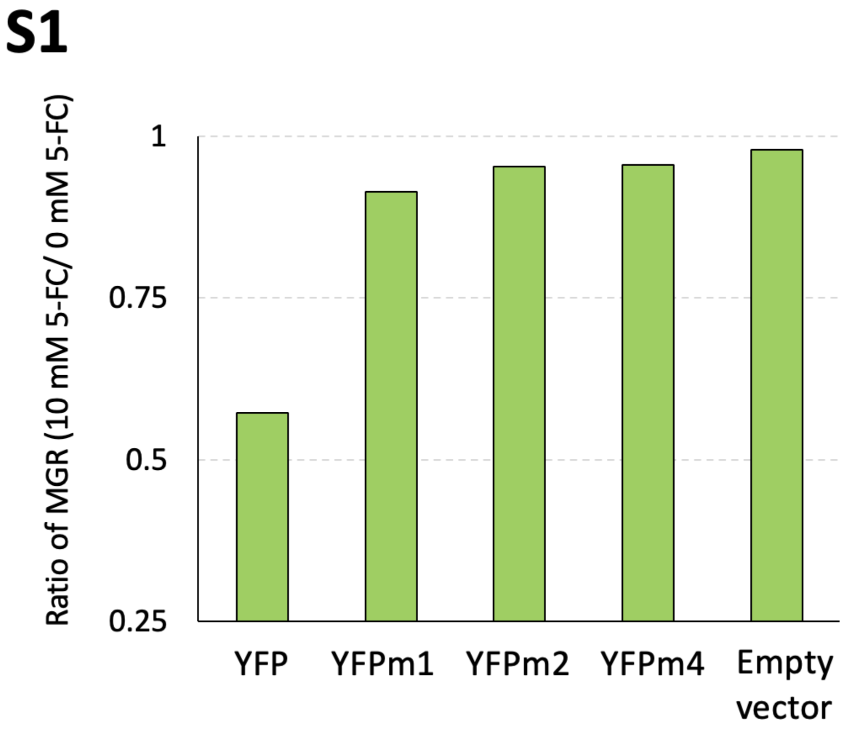

Figure S1. Ratio of the maximum growth rate of the yeast cells harboring pWF5-YFP, pWF5-YFPm1, pWF5-YFPm2, and pWF5-YFPm4 at aTc 500 nM in 10 mM 5-FC (test) and 0 mM 5-FC (control) conditions from **figure 1D**.

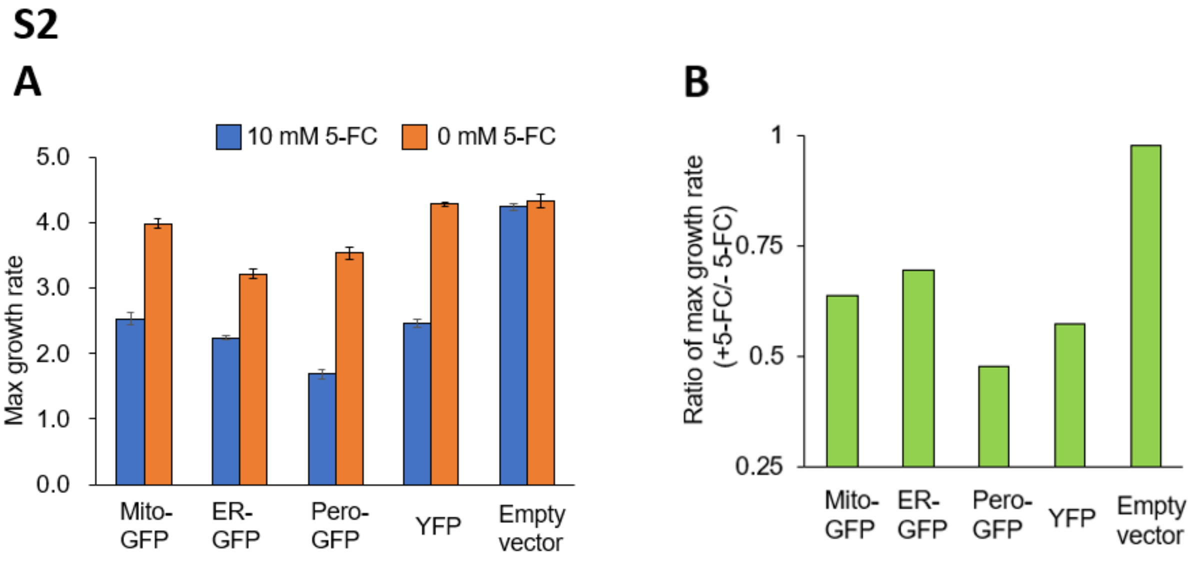

Figure S2. (A) Maximum growth rate of the yeast cells harboring the plasmids expressing Fcy1-Mito-GFP, Fcy1-ER-GFP, and Fcy1-Pero-GFP at aTc 500 nM in 10 mM 5-FC and 0 mM 5-FC conditions. (B) Ratio of the maximum growth rate of the yeast cells harboring the plasmids expressing Fcy1-Mito-GFP, Fcy1-ER-GFP

and Fcy1-Pero-GFP calculated from the results of (A).

S3

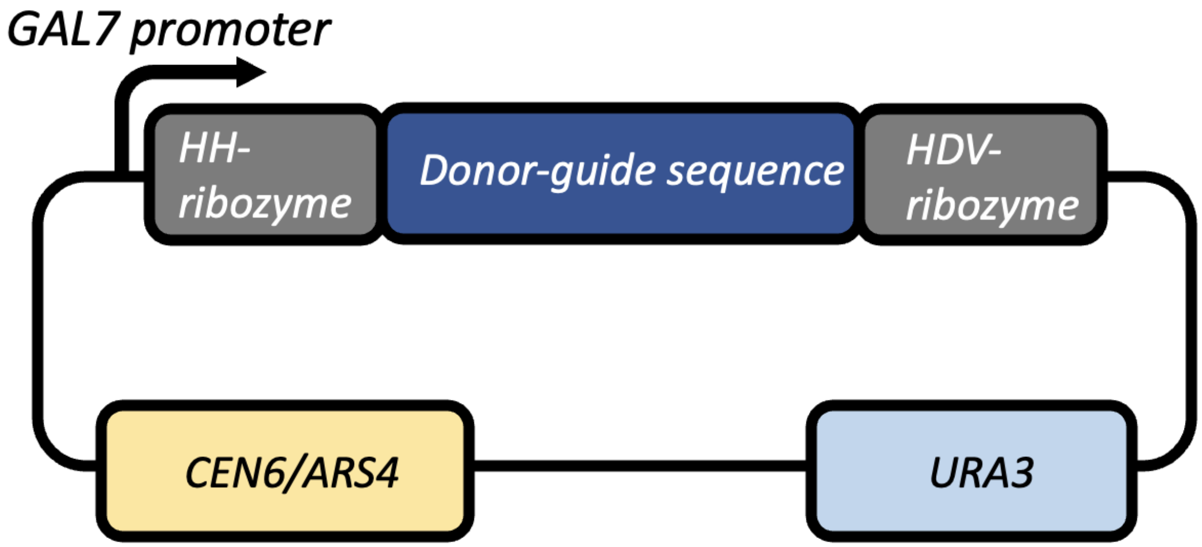

Figure S3. Design of pZS165BX, modified from plasmid pZS165 from the Fraser lab, used for the ligation of the guide/donor sequences in the creation of the Intra-*FCY1*-YFP library.

S4

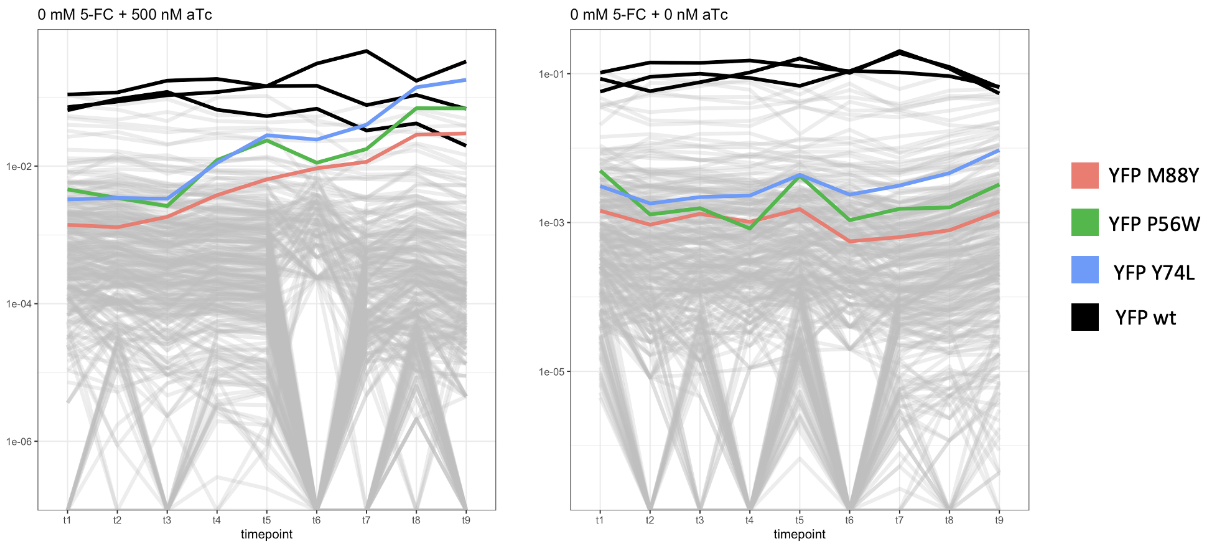

Figure S4. Frequency plots for the pooled competitive growth assays in 0mM 5-FC + 500 nM aTc and 0 mM 5-FC + 0 nM aTc. The YFP SM series strains are highlighted in color and the wildtype YFP strains are highlighted in black. All other strains are colored gray.

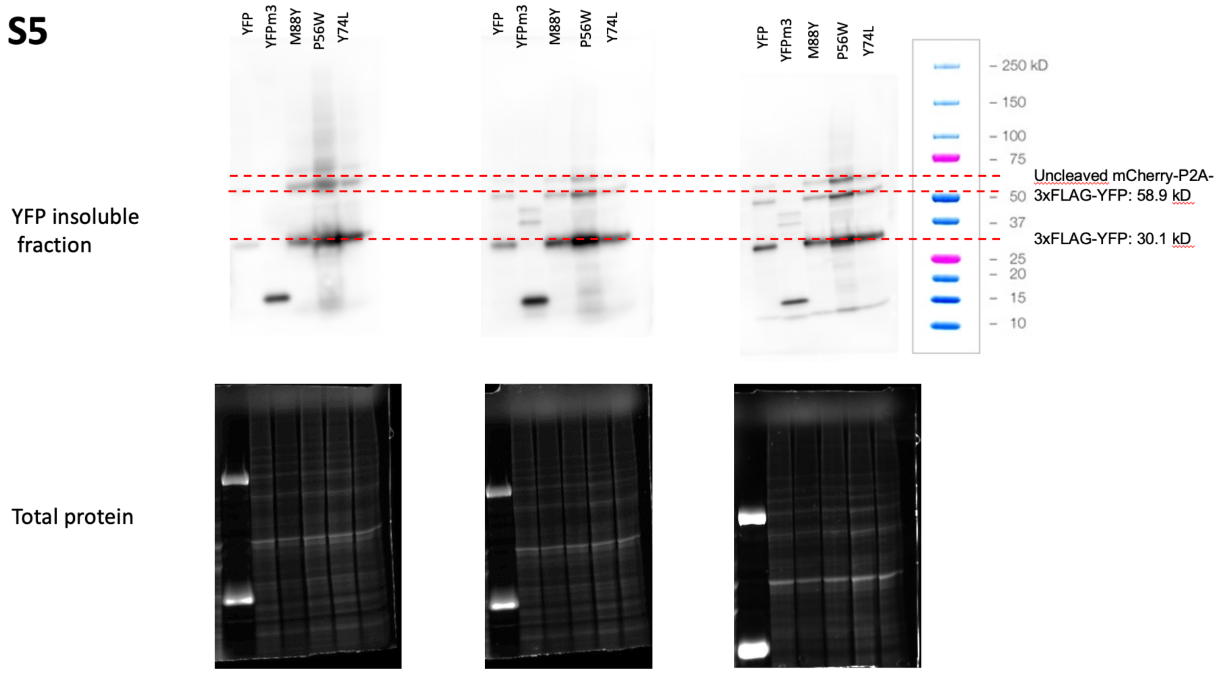

Figure S5. Western blots for YFP insoluble protein (top) and total protein (bottom) in wildtype YFP, YFPm3, and YFP SM series strains for 3 biological replicates. In all experiments, the YFPm3 band in the insoluble fraction (top) ran at a smaller size than the other bands, perhaps due to degradation despite having inhibited the proteasome.

S6

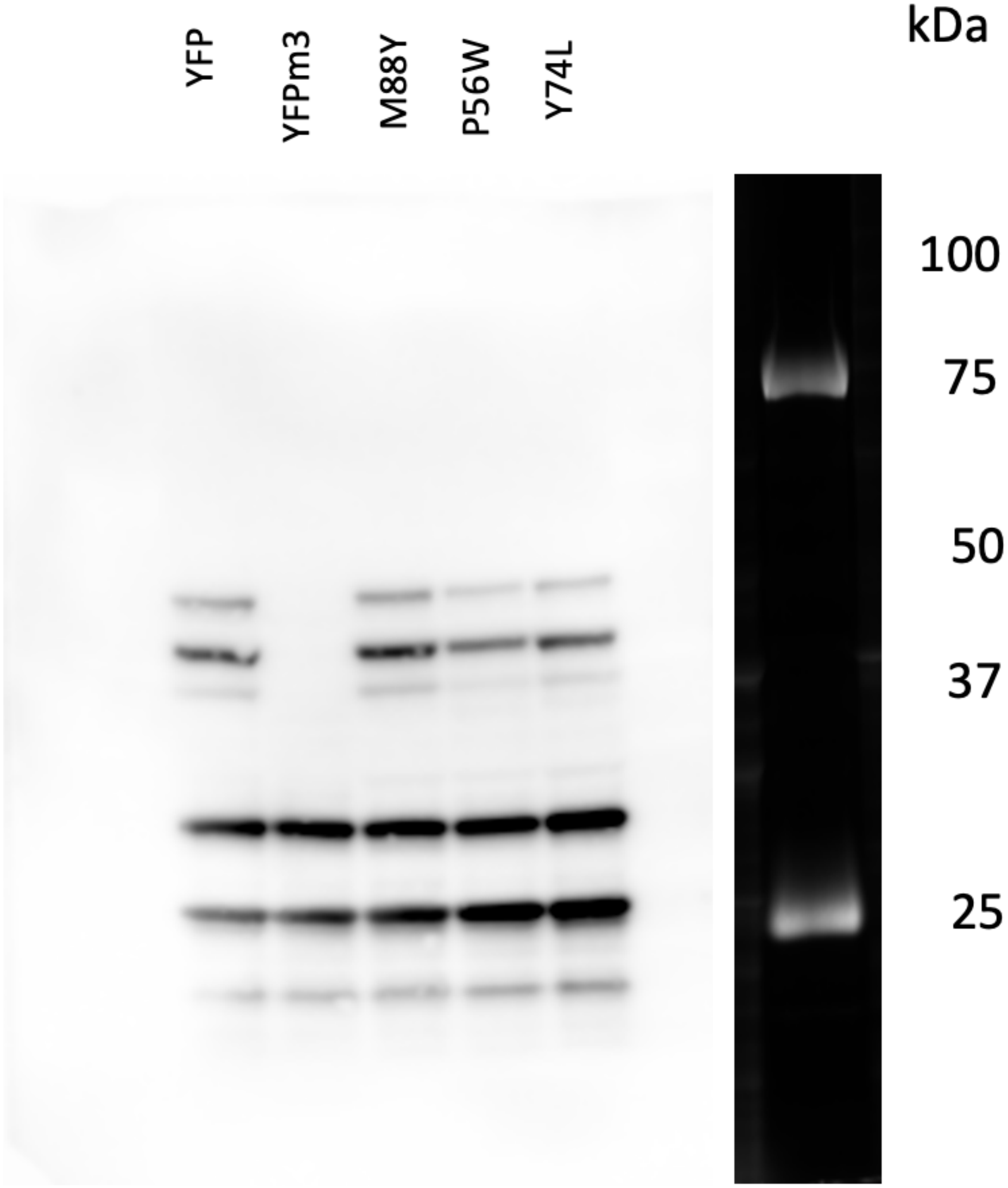

Figure S6. Western blot for mCherry soluble protein in wildtype YFP, YFPm3, and YFP SM series strains. The larger bands likely represent cases where the P2A was not cleaved so the mCherry remains linked to the YFP. This corresponds with the third column of **figure S5** and was used as an expression control. Since the band intensities of each protein in this blot are more uniform than those in **Figure S5**, it seems unlikely that the differences in band intensity in **Figure S5** are due to differences in expression levels of each YFP variant.

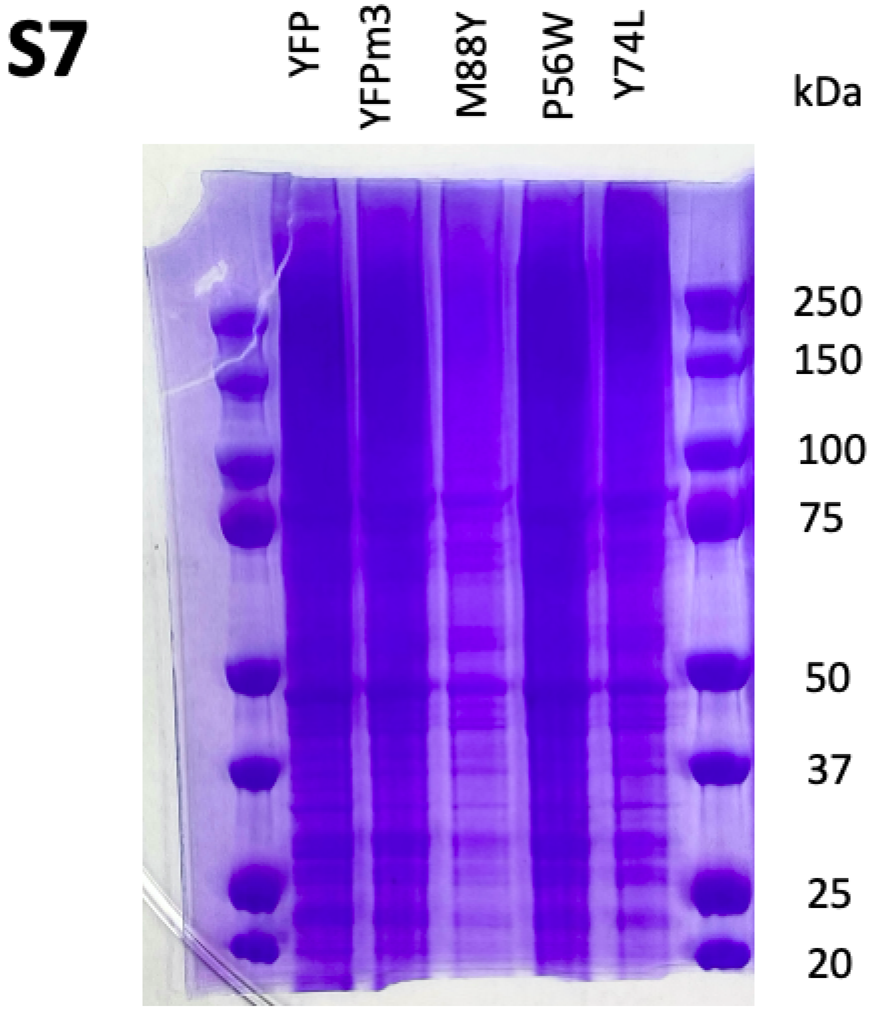

Figure S7. Coomassie stain for the insoluble protein fraction in wildtype YFP, YFPm3, and YFP SM series strains.
